# Comparative and phylogenetic analysis of the complete chloroplast genomes of *Uncaria* (Rubiaceae) species

**DOI:** 10.3389/fpls.2023.1271689

**Published:** 2023-12-22

**Authors:** Jiangpeng Dai, Qiaozhen Liu, Xingyuan Xu, Zhijie Tan, Yuexia Lin, Xiaoxia Gao, Shuang Zhu

**Affiliations:** ^1^ School of Biosciences and Biopharmaceutics, Guangdong Pharmaceutical University, Guangzhou, China; ^2^ School of Pharmacy, Guangdong Pharmaceutical University, Guangzhou, China

**Keywords:** *Uncaria*, chloroplast genome, taxonomy, codon usage bias, phylogenetic analysis

## Abstract

The genus *Uncaria* is famous for its high medicinal value. However, the high morphological similarities and unclear interspecific genetic relationships have posed challenges to the classification and identification of *Uncaria* species. Here, we newly sequenced six chloroplast genomes of *Uncaria* species: *U. hirsuta*, *U. rhynchophylla*, *U. rhynchophylloides*, *U. homomalla*, *U. sinensis*, and *U. lancifolia*. Comparisons among the chloroplast genomes of *Uncaria* species showed their conservation in structure, gene content, and order. Ten highly variable loci could be potentially used as specific molecular markers in the identification of *Uncaria* species. The third position of codons tended to use A/U base, and natural selection contributed more to the formation of codon usage bias in comparison to mutation pressure. Four genes (*rbc*L, *ndh*F, *rps*8, and *ycf*2) were detected to be subjected to positive selection. Phylogenetic analysis showed that the genus *Uncaria* was a monophyletic group, belonging to the tribe Naucleeae. Moreover, *U. sinensis* was not a variant of *U. rhynchophylla*. *U. rhynchophylloides* and *U. rhynchophylla* were not the same species. The results of the comparative and phylogenetic analysis provide valuable references for further research studies of classification, identification, breeding improvement, and phylogenetic relationships in *Uncaria* species.

## 1 Introduction

The genus *Uncaria* Schreb., in the family Rubiaceae, is primarily distributed in tropical Asia and Australia with 12 species distributed in China (Zhang et al., 2015). *Uncaria* species, commonly known as “Gouteng” in traditional Chinese medicine, have been extensively employed for the cure of various diseases over a considerable period of time (Zhang et al., 2015; Liu et al., 2023). Prior research studies have reported many pharmacological activities of *Uncaria* species, such as being antihypertensive, antiarrhythmic, anticonvulsant, sedative, antidepressant, and antithrombotic (Qin et al., 2021). The Chinese pharmacopoeia states that *U. rhynchophylla*, *U. macrophylla*, *U. hirsuta*, *U. sessilifructus*, and *U. sinensis* are medical *Uncaria* species (Chinese Pharmacopoeia Commission, 2020). The chemical compositions and bioactivities vary among different *Uncaria* species, which results in the divergence of curative effect (Liu et al., 2023). However, the high morphological similarities and the long-standing taxonomic controversies bring difficulties to the identification of *Uncaria* species.

The genus *Uncaria* was ever classified into the tribe Cinchoneae based on placentation characteristics (Ridsdale, 1978b). However, Razafimandimbison and Bremer (2001) supported the genus *Uncaria* classified into the tribe Naucleeae based on molecular and morphological data. Ridsdale (1978a) regarded *U. rhynchophylloides* as a synonym of *U. rhynchophylla*, but the point was doubted in some studies (Chen and Taylor, 2011; Turner, 2018; Zhu et al., 2018). Zhao et al. (2018) suggested that *U. sinensis* was possibly a variant of *U. rhynchophylla.* “*Flora of China*” accepted the species name of *U. homomalla* and *U. scandens*, but it presented that further study was needed to verify the separation of the two species due to the deficiency of obviously morphological differences (Chen and Taylor, 2011). DNA molecular markers, uninfluenced by morphological characters, have solved some taxonomic controversies such as the taxonomic position of the genus *Uncaria* (Razafimandimbison and Bremer, 2001; Manns and Bremer, 2010). Internal transcribed spacer (ITS) and ITS2 have shown excellent performance in the identification of *Uncaria* species (Zhang et al., 2015; Tang et al., 2016; Zhu et al., 2018; Liu et al., 2023). However, the low bootstrap support rates of some branches within the genus *Uncaria* were found in the phylogenetic trees (Zhang et al., 2015; Zhu et al., 2018; Liu et al., 2023), and the phylogenetic relationships were not completely resolved.

Chloroplast is a key organelle responsible for photosynthesis and numerous metabolic activities with independent genome and evolution routes (Li et al., 2020; Li et al., 2022). Chloroplast genome typically possesses conserved circular tetrad structure with the size range from 72 kb to 217 kb (Sheng et al., 2021b). In comparison with nuclear and mitochondrial genomes, chloroplast genome has the characteristics of low sequencing costs, large gene density, highly conserved structure, and moderate evolution rate (Huang et al., 2021). Despite relative conservation, mutation events of substitution, translocation, insertion, and deletion in chloroplast genomes still provide sufficient genetic information for exploring interspecific genetic relationships of plants (Sheng et al., 2021b). Moreover, the specific DNA fragments screened from coding and non-coding chloroplast genomic regions having different nucleotide substitution rate are suitable for phylogenetic research studies of different taxonomic levels (Zhang et al., 2022). Chen et al. (2022) investigated the phylogenetic relationships of five *Uncaria* species using chloroplast genomes, but the limited number of species did not adequately reveal the phylogenetic relationships of *Uncaria* species.

Here, the complete chloroplast genomes of six *Uncaria* species (*U. hirsuta*, *U. rhynchophylla*, *U. rhynchophylloides*, *U. homomalla*, *U. sinensis*, and *U. lancifolia*) were newly obtained by high-throughput sequencing. Combined with three previously released chloroplast genomes of *Uncaria* species, we did comprehensive comparative analysis of chloroplast genomes of *Uncaria* species. The objectives of the research were to 1) gain insights into chloroplast genome structural features of *Uncaria* species, 2) seek the high variable regions for use in *Uncaria* species identification, 3) explore the codon usage bias and affecting factors, and 4) elucidate the phylogenetic relationships of *Uncaria* and closely related species.

## 2 Materials and methods

### 2.1 Plant materials and DNA extraction

Six samples of *Uncaria* species (*U. hirsuta*, *U. rhynchophylla*, *U. rhynchophylloides*, *U. homomalla*, *U. sinensis*, and *U. lancifolia*) were collected from various regions of China and identified by Professor Changqing Zeng (Guangdong Pharmaceutical University, China) based on morphological traits (**Supplementary Table S1**). Genomic DNA of silica-dried leaves was extracted using modified cetyl trimethyl ammonium bromide protocol (Li et al., 2013).

### 2.2 Genome sequencing, assembly, and annotation

The high-quality genomic DNA was used to generate libraries with an average insert size of 350 bp and was sequenced on the Illumina NovoSeq 6000 platform (150-bp paired end). Fastp v0.23.0 was adopted to trim low-quality regions and adaptors of original sequencing data (Chen et al., 2018). GetOrganelle v1.7.5.0 was employed to assemble chloroplast genome sequences by selecting *U. sessilifructus* (ON243635) as seed sequence (Jin et al., 2020). Six sequences were annotated through Plastid Genome Annotator (PGA) and CPGAVAS2 tools with subsequently manual adjustment in Geneious Prime software (Kearse et al., 2012; Qu et al., 2019; Shi et al., 2019). Meanwhile, tRNAscan-SE v2.0.3 was employed to confirm transfer RNA (tRNA) genes (Lowe and Eddy, 1997). Three chloroplast genomes of *U. sessilifructus* (ON243635), *U. scandens* (ON243637), and *U. macrophylla* (ON243636) were downloaded for further comparative analysis. Subsequently, the homology among nine chloroplast genomes of *Uncaria* species was evaluated in Mauve program (Darling et al., 2004). In the end, the chloroplast genome maps were visualized using OGDRAW (Lohse et al., 2007).

### 2.3 Highly variable loci identification and simple sequence repeat analysis

Comparative chloroplast genomes of *Uncaria* species were carried out and visualized using mVISTA in shuffle-LAGAN mode with *U. sessilifructus* (ON243635) as a reference (Frazer et al., 2004). MAFFT v7.313 was used for sequences alignment, and nucleotide diversity (Pi) was subsequently calculated using DnaSP v6.12.03 with window length of 600 bp and step size of 200 bp (Librado and Rozas, 2009). MEGA v11.0.13 was used for calculation of interspecific genetic distance (Kumar et al., 2016). Simple sequence repeats (SSRs) were identified using MIcroSAtellite identification tool (MISA) with parameters set to 10, 5, 4, 3, 3, and 3 for mononucleotide, dinucleotide, trinucleotide, tetranucleotide, pentanucleotide, and hexanucleotide, respectively (Beier et al., 2017).

### 2.4 Codon usage bias

Common protein-coding sequences with more than 300 bp (Li et al., 2021a) were employed for codon usage analysis. Effective number of codons (ENC), relative synonymous codon usage (RSCU), and Guanine and Cytosine (GC) content at the third position of synonymous codons (GC3s) were calculated by CodonW v1.4.2 (http://codonw.sourceforge.net/). The online CUSP program (http://embossgui.sourceforge.net/demo/) was used to calculated GC content at the first (GC1), second (GC2), and third (GC3) position of codons. ENC plot was drawn with ENC value as ordinate and GC3s as abscissa to explore the factors affecting the codon usage. The standard curve was calculated according to the formula (Li et al., 2021a): ENC_expected_ = 2 + GC3s + 29/[GC3s^2^ + (1 − GC3s)^2^]. Using GC12 (the average value of GC1 and GC2) as ordinate and GC3 as abscissa to make the neutral plot could investigate the degree of impact of mutation pressure and natural selection on codon usage bias (Chakraborty et al., 2020; Wang et al., 2022b). The slope of the regression line close to zero showed that codon usage bias was primarily influenced by natural selection, whereas the slope of the regression line close to one indicated that the codon usage bias was primarily influenced by mutation pressure (Chakraborty et al., 2020; Wang et al., 2022b).

### 2.5 Adaptive evolution analysis

The common protein-coding genes (PCGs) were aligned by MAFFT package performed in PhyloSuite v1.2.2 with codon alignment pattern (Zhang et al., 2020). The non-synonymous (dN) and synonymous (dS) substitution and their ratio dN/dS were calculated using DnaSP v6.12.03 software (Librado and Rozas, 2009). The ratio dN/dS > 1, dN/dS = 1, and dN/dS < 1 indicated positive, neutral, and negative selection, respectively (Liu et al., 2021). Moreover, the site model was employed to identify potentially positive selection sites using the CODEML algorithm performed in EasyCodeML v1.4 (Gao et al., 2019). Bayes Empirical Bayes method was used to calculate the posterior probabilities for amino acid sites that were potentially under positive selection. The logarithmic likelihood value of four pair of models (M0 vs.M3, M1a vs.M2a, M7 vs.M8, and M8a vs.M8) was calculated by likelihood ratio test and its statistical significance.

### 2.6 Phylogenetic analysis

The chloroplast genomes of nine *Uncaria* species, 40 Rubiaceae species, and Gelsemium sempervirens (outgroup) were adopted for phylogenetic analysis (**Supplementary Table S2).** Two datasets of the complete chloroplast genome sequences and concatenation common protein-coding sequences were adopted for construction of phylogenetic trees, respectively. MAFFT v7.313 was used for sequences alignment (Katoh and Standley, 2013). The poorly aligned regions were removed by trimAl v1.2 (Capella-Gutiérrez et al., 2009). Phylogenetic trees were constructed through neighbor joining (NJ), maximum likelihood (ML), and Bayesian inference (BI) methods. The best-fitting nucleotide substitution models were selected by ModelFinder (Kalyaanamoorthy et al., 2017). NJ analysis was carried out using MEGA v11.0.13 with 1,000 bootstrap replicates. ML analysis was conducted using IQ-TREE v1.6.8 with 1,000 bootstrap replicates (Nguyen et al., 2015). BI analysis was conducted using MrBayes v3.2.6 (Ronquist and Huelsenbeck, 2003), and the setting parameters followed the study of Zhang et al. (2022).

## 3 Results

### 3.1 Features of chloroplast genome

**Figure 1:**
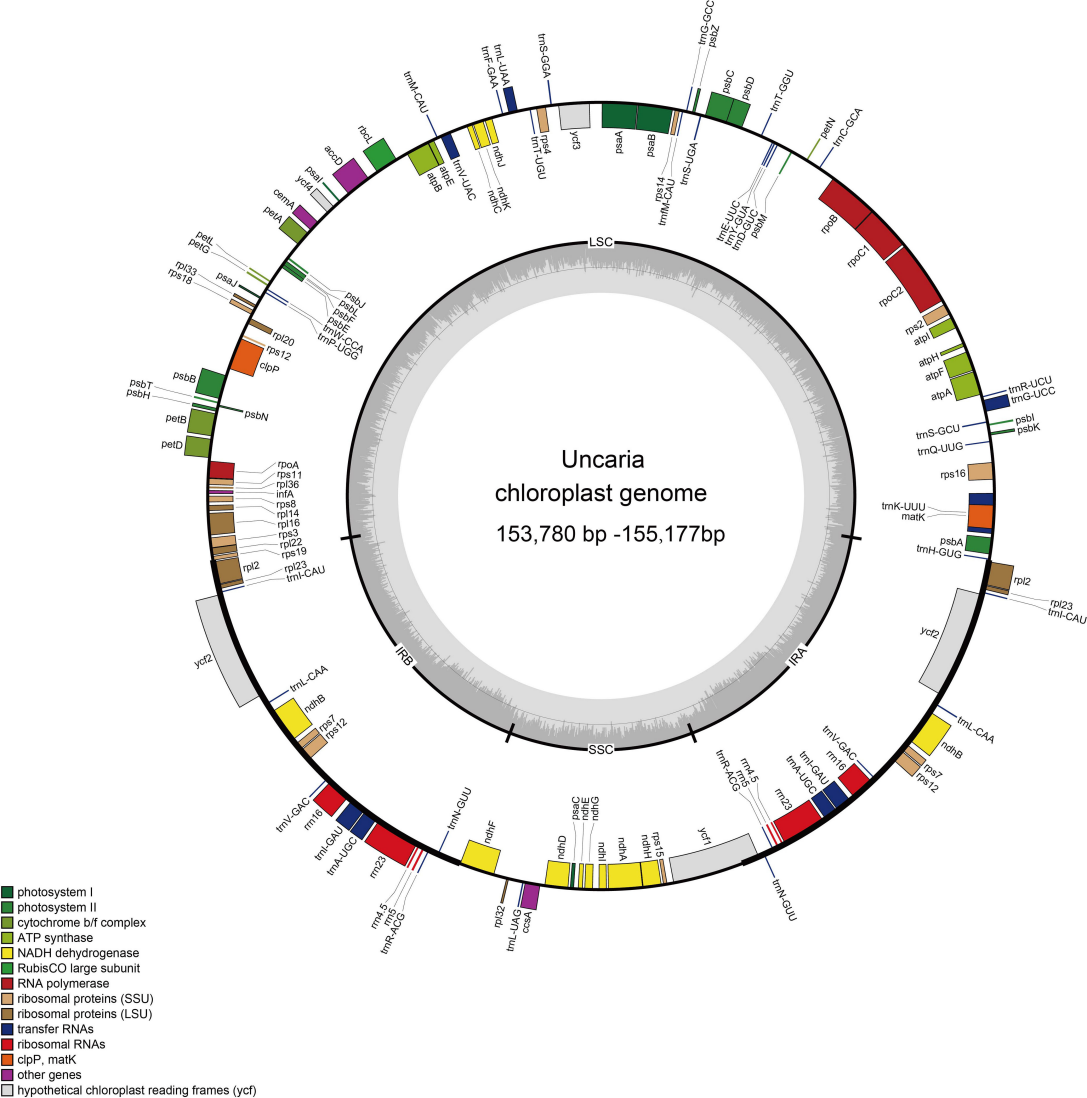
The chloroplast genome map of *Uncaria* species.

**Figure 2:**
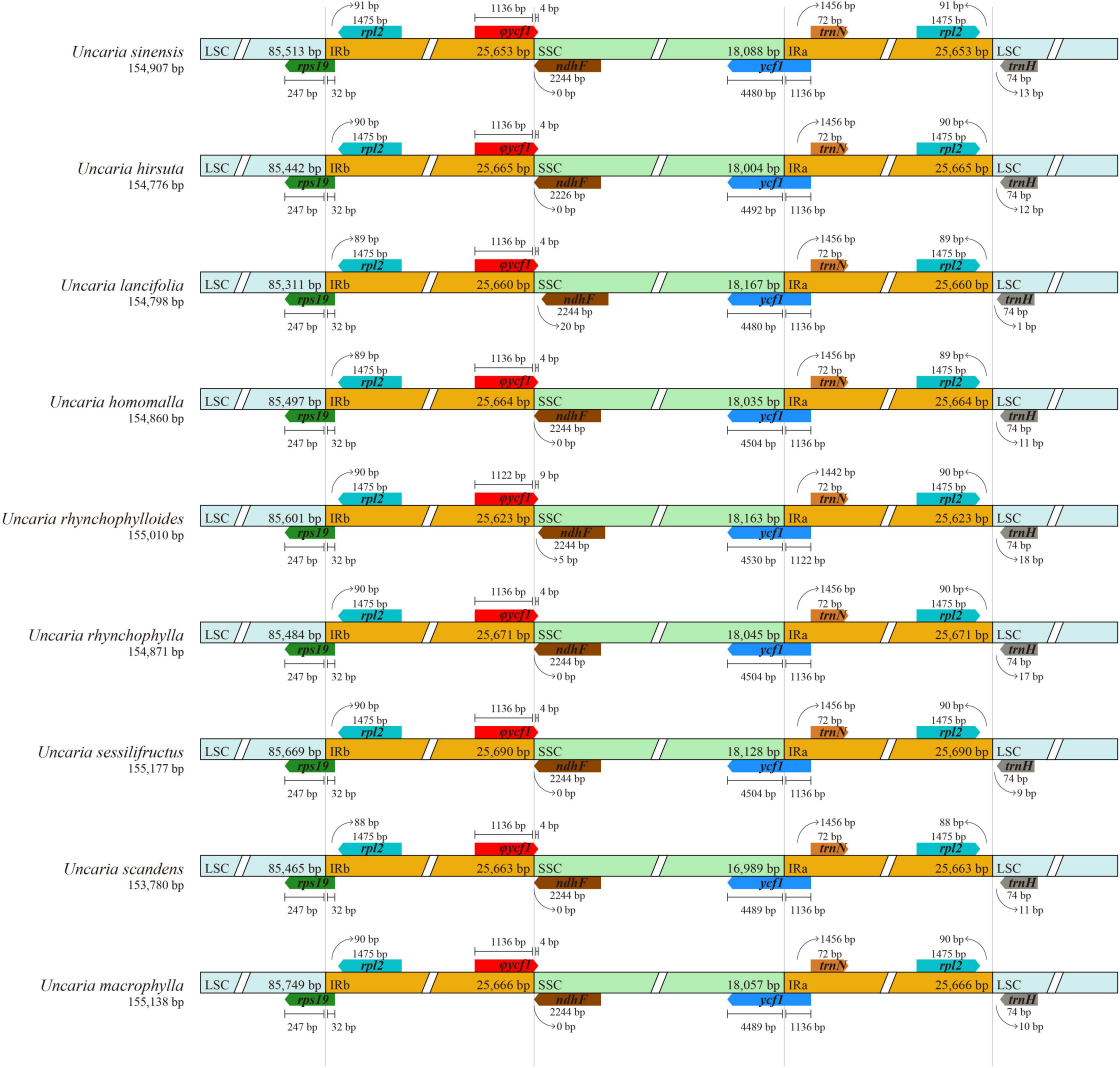
Comparison of LSC, SSC, and IR boundary regions among the chloroplast genomes of *Uncaria* species. φ indicates a pseudogene.

The raw data of six *Uncaria* species in this study were in the range of 4.53 Gb to 11.92 Gb (**Supplementary Table S3**). After trimming low-quality regions and adaptors, 4.46 Gb to 11.45Gb clean data were used for assembly. To estimate the quality and reliability of assembly results, clean reads were mapped to the assembled chloroplast genomes. There were no low read coverage regions detected in assembled chloroplast genomes of *Uncaria* species (**Supplementary Figure S1**). The nine chloroplast genomes of *Uncaria* species ranged in length from 153,780 bp (*U. scandens*) to 155,177 bp (*U. sessilifructus*). The chloroplast genomes of the nine *Uncaria* species exhibited a typically circular tetrad structure comprising a large single-copy (LSC) region, a small single-copy (SSC) region, and two inverted repeat (IR) regions (**Figure 1**). As shown in **Table 1**, the lengths of LSC regions ranged from 85,311 bp (*U. lancifolia*) to 85,749 bp (*U. macrophylla*), and the lengths of SSC regions varied from 16,989 bp (*U. scandens*) to 18,167 bp (*U. lancifolia*). The lengths of IR regions had inconspicuous variation among *Uncaria* species and ranged from 25,623 bp (*U. rhynchophylloides*) to 25,690 bp (*U. sessilifructus*). Each of the nine chloroplast genomes possessed 129 to 130 genes, comprising 84 to 85 PCGs, 37 tRNA genes, and eight ribosomal RNA (rRNA) genes (**Table 1**; **Supplementary Table S4**). Compared with other *Uncaria* species, *U. scandens* lost *ccs*A gene in its chloroplast genome, which caused a reduction in the length of chloroplast genome. In each chloroplast genome of *Uncaria* species (**Supplementary Table S4**), 18 genes containing intron were found, and there were three genes (*rps*12, *ycf*3, and *clp*P) with two introns, whereas the others possessed one intron. Mauve alignment results showed good synteny of the nine chloroplast genomes, and gene rearrangements were not observed (**Supplementary Figure S2**). Furthermore, the region borders and nearby genes of the chloroplast genomes were compared (**Figure 2**). Among the nine *Uncaria* species, the *ycf*1 gene spanned the IRa/SSC junction with the length of 4,480 bp to 4,530 bp located in SSC region. However, another short *ycf*1 fragment situated at SSC/IRb border was detected as pseudogene, which approximately lost a fragment of 4,500 bp in length compared with complete *ycf*1 gene.

**Table 1:** Summary of the chloroplast genome features of *Uncaria* species.

Species	LSC length (bp) GC (%)	IR length (bp) GC (%)	SSC length (bp) GC (%)	Total length (bp) GC (%)	Gene	tRNA	rRNA	PCGs	Accession number
*U. hirsuta*	85,442 35.55	25,665 43.18	18,004 31.68	154,776 37.63	130	37	8	85	OQ679827
*U. homomalla*	85,497 35.55	25,664 43.18	18,035 31.81	154,860 37.64	130	37	8	85	OQ679828
*U. lancifolia*	85,311 35.54	25,660 43.22	18,167 31.51	154,798 37.61	130	37	8	85	OQ679829
*U. macrophylla*	85,749 35.40	25,666 43.19	18,057 31.55	155,138 37.53	130	37	8	85	ON243636
*U. rhynchophylla*	85,484 35.57	25,671 43.17	18,045 31.75	154,871 37.65	130	37	8	85	OQ679830
*U. rhynchophylloides*	85,601 35.51	25,623 43.21	18,163 31.61	155,010 37.60	130	37	8	85	OQ679831
*U. scandens*	85,465 35.55	25,663 43.18	16,989 31.69	153,780 37.67	129	37	8	84	ON243637
*U. sessilifructus*	85,669 35.47	25,690 43.16	18,128 31.49	155,177 37.55	130	37	8	85	ON243635
*U. sinensis*	85,513 35.57	25,653 43.20	18,088 31.66	154,907 37.64	130	37	8	85	OQ679832

### 3.2 Sequence divergence and mutational hotspots

**Figure 3:**
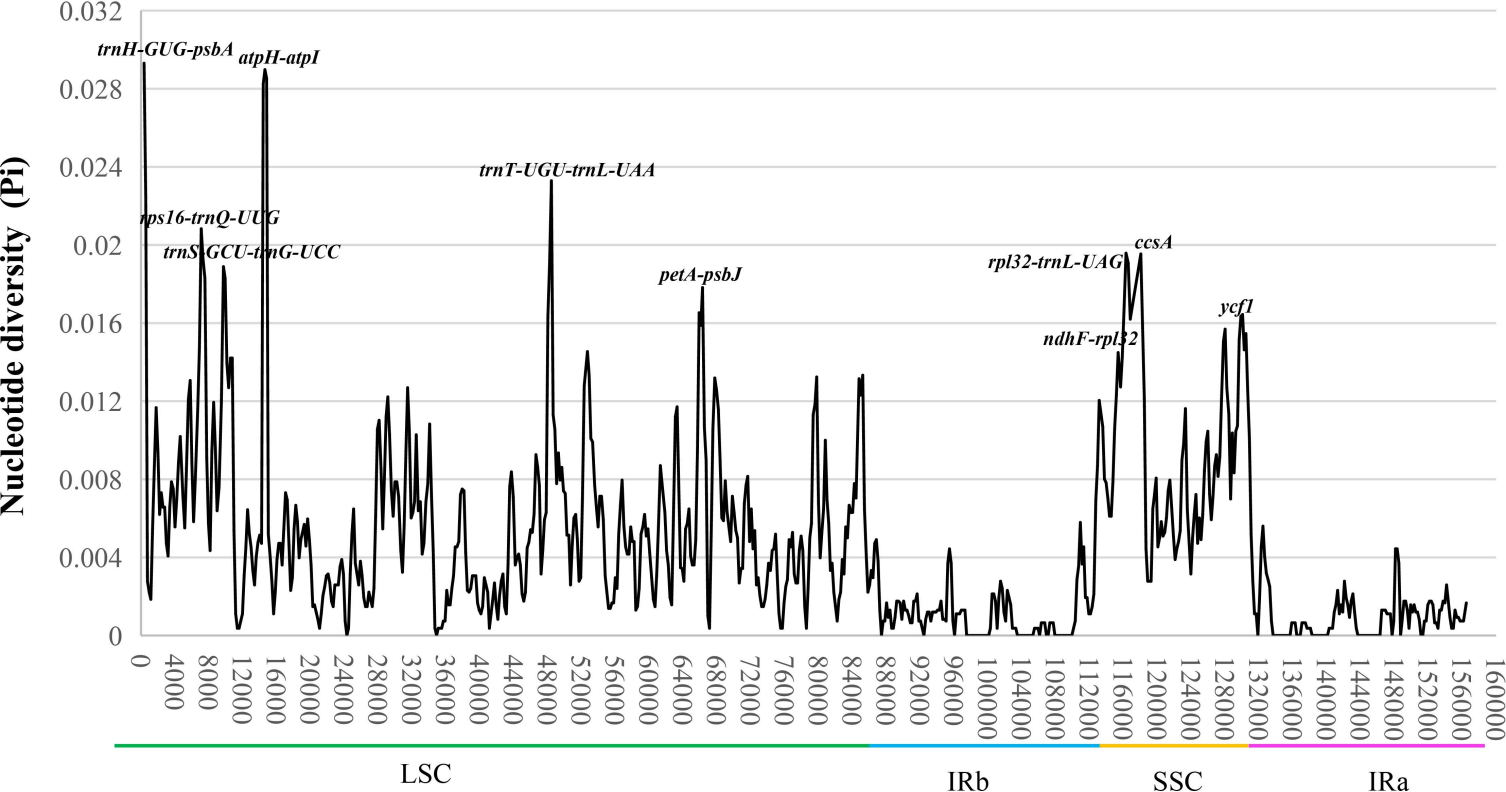
The nucleotide diversity (Pi) of the chloroplast genomes of nine *Uncaria* species. Ten highly variable loci (Pi > 0.015) are marked out.

The mVISTA result exhibited a high sequence identity among the chloroplast genomes of nine *Uncaria* species, and a majority of highly variation regions were founded in non-coding regions (**Supplementary Figure S3**). The nucleotide diversity (Pi) value ranged from 0 to 0.2931 with the average of 0.004525 (**Figure 3**). The average Pi value of IR regions (0.0009849) was much lower than that in LSC (0.005740) and SSC regions (0.009168), which indicated lower sequence discrepancy appearing in IR regions. A total of 10 highly variable loci were found in chloroplast genomes of *Uncaria* species. Among them, eight highly variable loci (*trn*H-GUG-*psb*A, *atp*H-*atp*I, *trn*T-UGU-*trn*L-UAA, *rps*16-*trn*Q-UUG, *rpl*32-*trn*L-UAG, *trn*S-GCU-*trn*G-UCC, *pet*A-*psb*J, and *ndh*F-*rpl*32) were found in non-coding regions, and two highly variable loci (*ccs*A and *ycf*1) were detected in coding regions. In addition, we calculated interspecific genetic distance of *Uncaria* species. On the basis of the complete chloroplast genome sequences, the pairwise genetic distances varied from 0.009 (*U. scandens*–*U. homomalla*) to 0.064 (*U. sessilifructus*–*U. sinensis*), with the average of 0.046 (**Supplementary Table S5**). On the basis of common protein-coding sequences of chloroplast genome, the pairwise genetic distances ranged from 0.007 (*U. scandens*–*U. homomalla*) to 0.0052 (*U. sessilifructus*–*U. sinensis*), with the average of 0.034 (**Supplementary Table S6**).

### 3.3 Simple sequence repeat analysis

**Figure 4:**
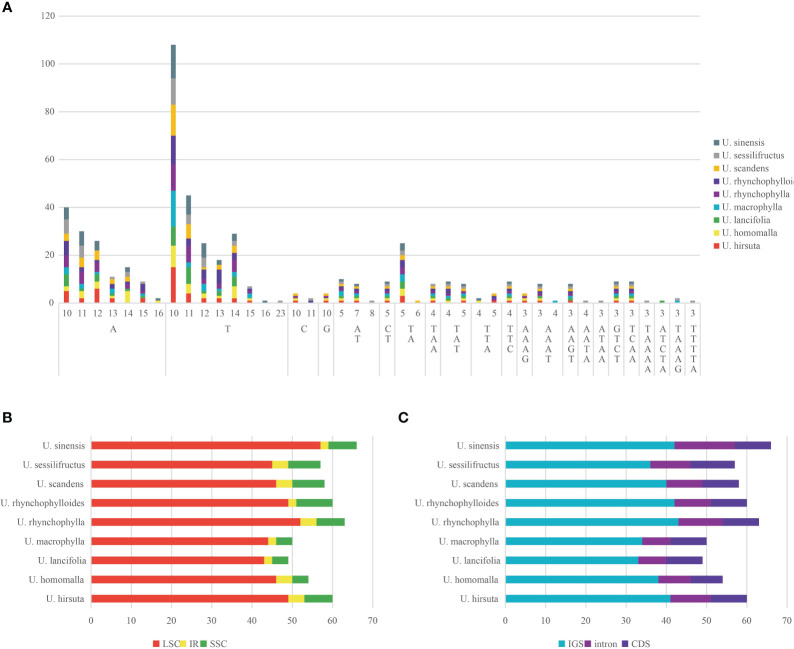
Analysis of SSRs in the chloroplast genomes of *Uncaria* species. **(A)** The type and number of SSRs. **(B)** The number of SSRs distributed in LSC, SSC, and IR regions. **(C)** The number of SSRs distributed in coding sequence (CDS), intron, and intergenic spacer (IGS) regions.

The number of SSRs detected in each chloroplast genome varied from 49 (*U. lancifolia*) to 66 (*U. sinensis*), and most of SSRs were mononucleotide repeats (68.00%–78.79%), followed by dinucleotide repeats (8.77%–14.00%) (**Figure 4A**). A large proportion of SSRs were composed of A/T base (83.33%–89.71%). Pentanucleotide repeats were only detected in *U. lancifolia*, *U. macrophylla*, and *U. sessilifructus*. There was no hexanucleotide repeat found in *Uncaria* species. Moreover, SSRs were principally distributed in LSC regions (78.95%–87.76%), followed by SSC regions (7.41%–15.00%) and IR regions (3.03%–7.41%) (**Figure 4B**). Moreover, a large proportion of SSRs were located in intergenic spacer (IGS) regions (63.16%–70.37%) (**Figure 4C**). In spite of low proportion, SSRs were also detected in intron regions (14.00%–22.73%) and coding regions (13.64%–19.30%).

### 3.4 Codon usage bias

**Figure 5:**
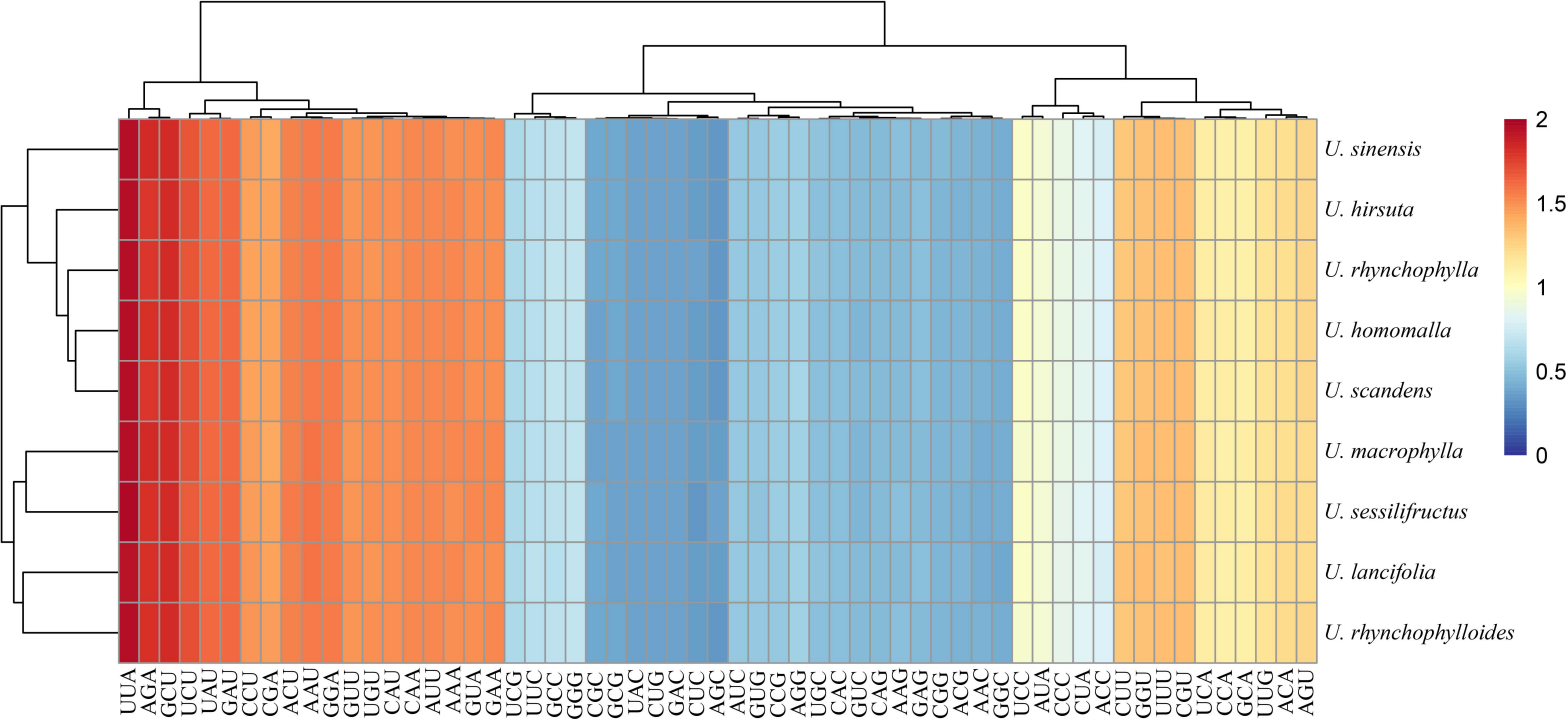
RSCU value among the chloroplast genomes of *Uncaria* species.

The GC content at three codon positions (GC1, GC2, and GC3) of the nine *Uncaria* species was less than 50% (**Table 2**). The correlation analysis exhibited that GC1, GC2, and GC12 were not significantly correlated with ENC, whereas GC3 and GC3s were significantly correlated with ENC (**Supplementary Table S7**). The RSCU values among *Uncaria* species were very similar (**Figure 5**), and UUA codon encoding Leucine had the maximum RSCU value (1.93–1.96). Each chloroplast genome possessed 29 high-frequency codons (RSCU > 1), and there were 16 codons ended with U base, 12 codons ended with A base, and only one codon ended with G base. However, among 30 low-frequency codons (RSCU < 1), there were only two codons ended with A base, and the others were ended with G/C base. The third position of codon tended to use A/U base in chloroplast genomes of *Uncaria* species.

**Table 2:** Main parameters of codon usage bias.

Species	GC1 (%)	GC2 (%)	GC3 (%)	GC3s (%)	ENC
*U. hirsuta*	47.39	39.46	28.32	25.14	47.11
*U. homomalla*	47.39	39.44	28.38	25.21	47.13
*U. lancifolia*	47.37	39.47	28.31	25.14	47.20
*U. macrophylla*	47.33	39.44	28.17	24.99	47.04
*U. rhynchophylla*	47.40	39.47	28.40	25.23	47.25
*U. rhynchophylloides*	47.39	39.46	28.32	25.14	47.11
*U. scandens*	47.41	39.44	28.37	25.20	47.20
*U. sessilifructus*	47.17	39.37	28.29	25.08	47.25
*U. sinensis*	47.42	39.45	28.42	25.26	47.29

The ENC plot exhibited that the point distribution patterns of coding sequences were similar among *Uncaria* species (**Supplementary Figure S4**). Some coding sequences were distributed close to the expected curve, whereas most of coding sequences were distributed far away from the expected curve. The results revealed that natural selection also affected the codon usage preference of chloroplast genomes of *Uncaria* species, other than mutation pressure, and their strength varied among genes. The neutral plot could further estimate the strength of mutation pressure and natural selection driving the formation of codon usage bias (**Supplementary Figure S5**). GC3 was not significantly correlated with GC12, and the slope of the regression line was much lower than 0.5, which implied that natural selection was the dominant factor affecting codon usage bias of *Uncaria* chloroplast genomes.

### 3.5 Adaptive evolution

**Figure 6:**
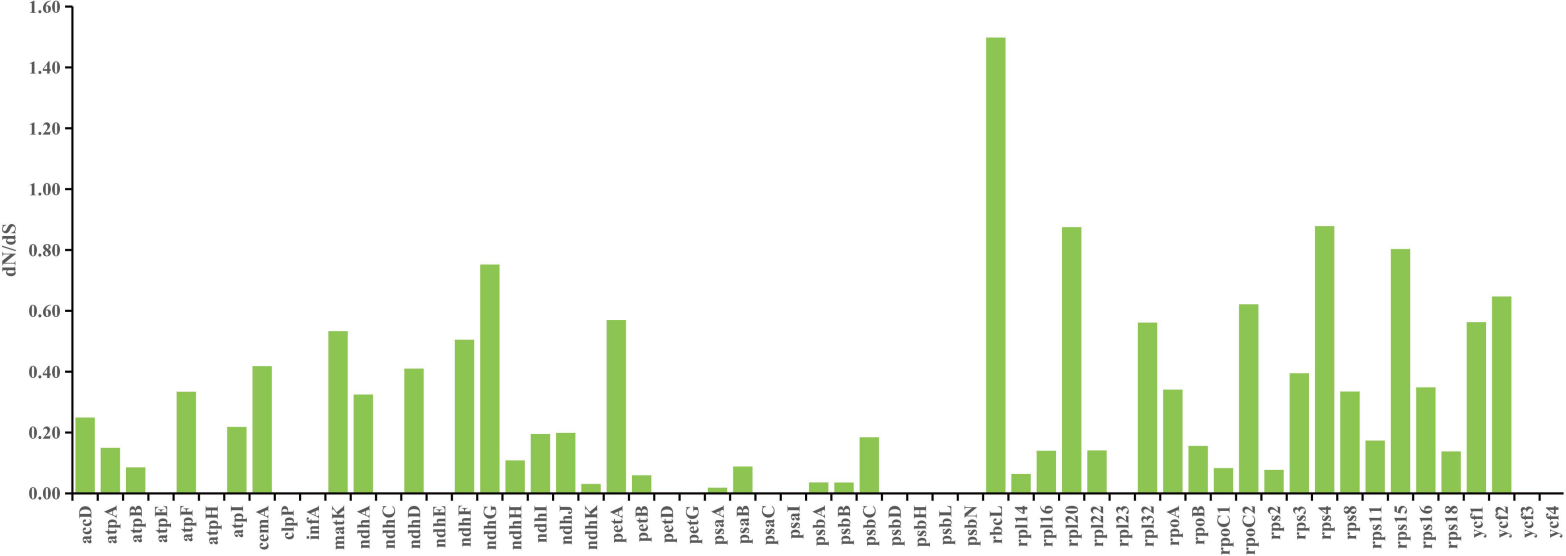
The dN/dS value of common protein-coding genes calculated by DnaSP.

**Figure 7:**
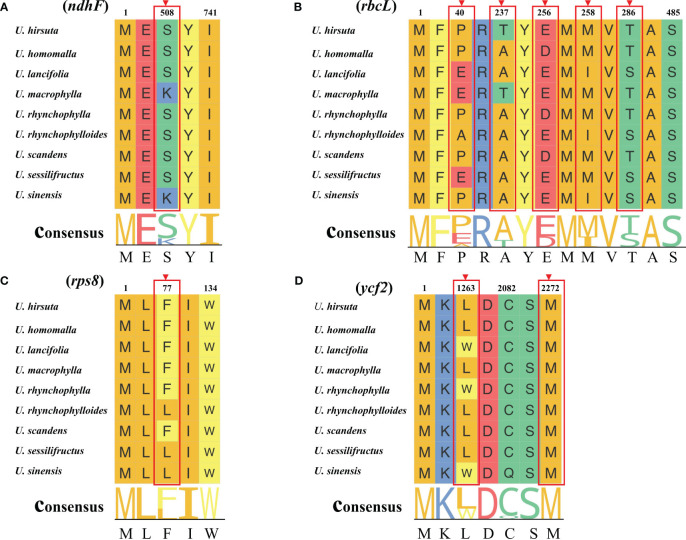
Four positive genes detected by site model. The positive selection sites with the posterior probabilities > 0.95 are marked out. **(A)** *ndhF* gene. **(B)** *rbcL* gene. **(C)** *rps8* gene **(D)** *ycf2* gene.

As shown in **Figure 6**, the *rbc*L gene was the only gene with dN/dS value higher than 1, and dN/dS values of other genes were lower than 1, which indicated that most of the PCGs in chloroplast genomes of *Uncaria* species were mainly subjected to negative selection in the process of evolution. In addition, site model was employed to detect potentially positive selection sites of each gene. Four genes were identified to have positive selection sites (**Figure 7**; **Supplementary Table S8**). Among them, the *rbc*L gene possessed five positive selection sites, the *ycf*2 gene possessed two positive selection sites, and *ndh*F and *rps*8 genes separately contained one positive selection site.

### 3.6 Phylogenetic analysis

**Figure 8:**
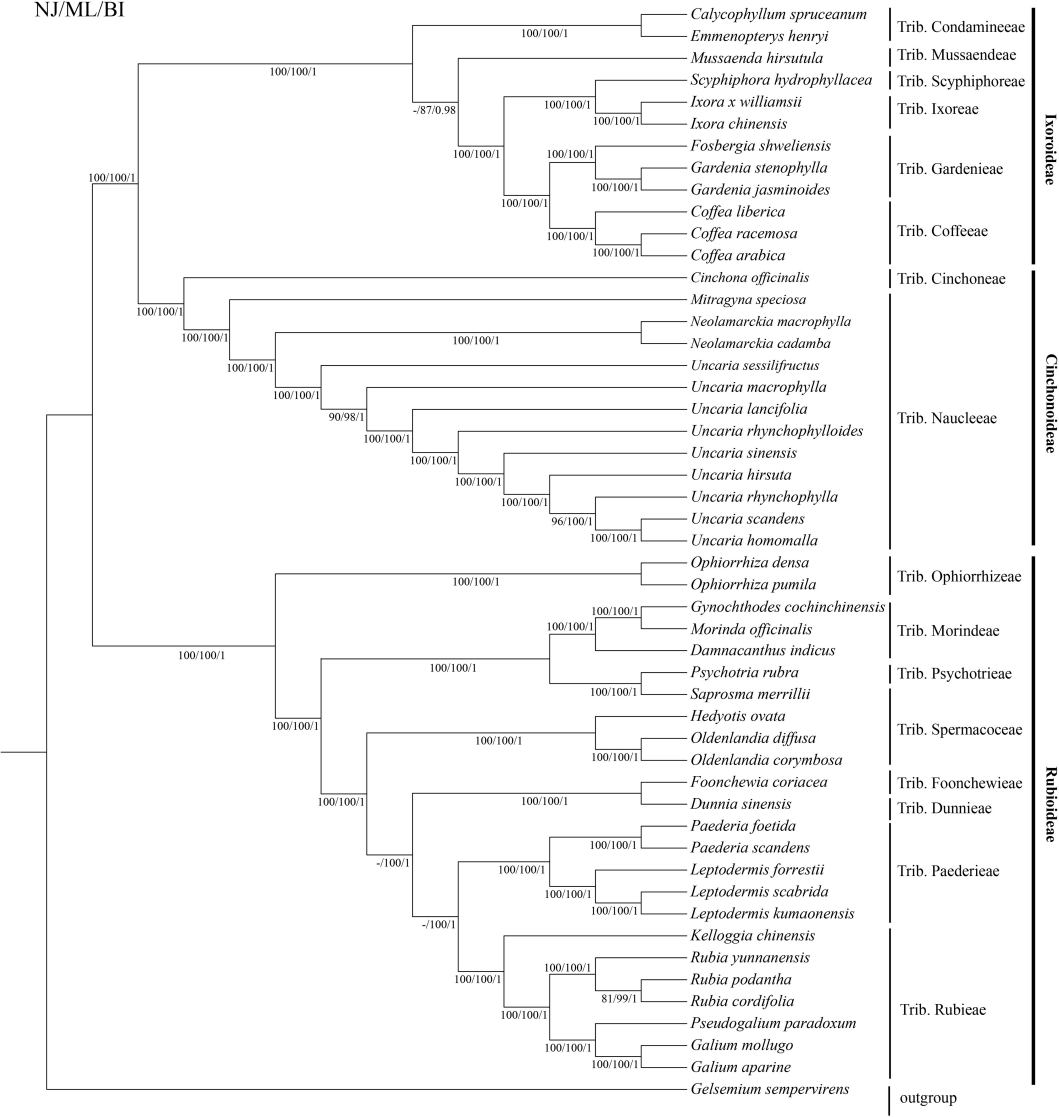
Phylogenetic tree of *Uncaria* and closely related species based on the complete chloroplast genome sequences.

We used the complete chloroplast genome sequences and common protein-coding sequences to reconstruct phylogenetic trees of *Uncaria* and Rubiaceae species (**Figure 8**; **Supplementary Figure S6**). The topologies of the two phylogenetic trees were highly similar, and all branches within the genus *Uncaria* had high values of neighbor joining bootstrap support (NJBS), maximum likelihood bootstrap support (MLBS), and Bayesian inference posterior probability (BIPP). In general, Rubiaceae species were classified into three well-supported large clades (NJBS, 100%/100%; MLBS, 100%/100%; and BIPP, 1/1), and Ixoroideae clade was a sister to Cinchonoideae clade (NJBS, 100%/100%; MLBS, 100%/100%; and BIPP, 1/1). *Uncaria* species formed a well-supported monophyletic clade (NJBS, 100%/100%; MLBS, 100%/100%; and BIPP, 1/1) that is a sister to the clade made up of *Neolamarckia* species (NJBS, 100%/100%; MLBS, 100%/100%; and BIPP, 1/1). Within the *Uncaria* clade, *U. sessilifructus* occupied the basal position, whereas *U. macrophylla* was placed in the position between *U. sessilifructus* and the clade composed of remaining *Uncaria* species (NJBS, 90%/97%; MLBS, 98%/91%; and BIPP, 1/0.99). *U. homomalla* and *U. scandens* were clustered as the youngest branch of *Uncaria* (NJBS, 100%/98%; MLBS, 100%/99%; and BIPP, 1/1) and then clustered with *U. rhynchophylla* (NJBS, 96%/99%; MLBS, 100%/100%; and BIPP, 1/1). The phylogenetic analysis results indicated that each clade within *Uncaria* was well supported and clustered.

## 4 Discussion

In this study, the nine chloroplast genomes of *Uncaria* species exhibited a conserved circular tetrad structure, which was also found in other Rubiaceae species (Zhang et al., 2021; Amenu et al., 2022). Owing to the loss of *ccs*A gene, the length of SSC region of *U. scandens* was greatly lower than other *Uncaria* species. The *ccs*A gene encodes a protein to mediate heme attached to C-type cytochrome (Li et al., 2021b). The pseudogenization and the loss of *ccs*A gene were found in most heterotrophic plants but were rarely found in autotrophic plant (Wicke and Naumann, 2018; Li et al., 2021b). Further studies were needed to judge whether the *ccs*A gene was transferred to nuclear genome of *U. scandens*. Expansion and contraction of IR regions are closely associated with the length change of chloroplast genome, which can cause gene loss, gene duplication, gene rearrangement, and pseudogenization in some angiosperms (Li et al., 2020). Because of the *ycf*1 gene spanning across SSC/IRa boundary, an incomplete fragment of the *ycf*1 gene appeared at IRb/SSC boundary in each chloroplast genome of *Uncaria* species. Although the fragment had intact open reading frames, it was identified as a pseudogene in many prior studies (Sheng et al., 2021b; Zhang et al., 2021; Bai et al., 2023). The gene distributions of region boundaries were highly similar among the chloroplast genomes of *Uncaria* species, and minor contraction and expansion of IR regions were found.

In spite of relatively low sequence variation levels among the chloroplast genomes of *Uncaria* species, 10 highly variable loci were detected, which could serve as potentially specific DNA molecular markers in the identification and phylogenetic research studies of *Uncaria* species. Most of highly variable loci were located in non-coding regions implying that non-coding regions mutated more quickly than coding regions. Furthermore, the IR regions exhibited higher conservation than SSC and LSC regions, and no highly variable loci were detected in IR regions except for *ycf*1. The higher conservation may be associated with copy correction between IR regions through gene conversion and replication (Liu et al., 2022).

SSRs, extensively distributed in the eukaryotic chloroplast genome, were regarded as effective molecular markers applicable to the studies of species identification, individual genetic difference. and population evolution (Li et al., 2020; Wu et al., 2021). The size, type, and number of SSRs varied among different *Uncaria* species. A large proportion of SSRs were mononucleotide repeats and biased toward A/T base, which were similar to the common SSR characteristics of chloroplast genomes in angiosperms (Li et al., 2020). Moreover, the non-coding and single-copy regions were the main distribution regions of SSRs, probably associated with the high-nucleotide polymorphism of these regions.

Codon usage bias means the frequency discrepancy of synonymous codon usage in organisms (Sheng et al., 2021a). It is universally assumed that codon usage bias can reveal the origin, evolution, and mutation modes of genes or species and have a significant impact on protein expression and gene function (Wang et al., 2020). The GC content of different codon position of the nine *Uncaria* species was lower than 50%, and most of high-frequency codons were ended with A/U base, which showed a high preference to use A/U-ending codons in chloroplast genomes of *Uncaria* species. Prior studies demonstrate that the nuclear genes preferred to use A/U-ending and G/C-ending codons in dicots and monocots, respectively (Zhang et al., 2012). However, A/U-ending codons were generally found as preference codons in chloroplast genomes (Nie et al., 2013). Furthermore, there were no codons detected with an RSCU value higher than 2, and the ENC values were higher than 45, implying a weak codon usage preference of chloroplast genomes of *Uncaria* species. Many factors influenced codon usage bias, but natural selection forces and mutation pressure were universally regarded as the major factors, which were extensively used to explain intraspecific and interspecific codon usage variation (Zhang et al., 2012; Sheng et al., 2021a). Mutation pressure can act on nucleotide composition preference by shuffling G/C and A/T pairs. Natural selection can cause the codon preference by maximizing efficiency of protein production in highly expressed genes (Duan et al., 2021). The results of ENC plot and neutral plot indicated that mutation pressure and natural selection were the important factors affecting the codon usage bias of chloroplast genomes of *Uncaria* species, especially the natural selection made the primary contribution.

Although a majority of PCGs in chloroplast genomes of *Uncaria* species were mainly subjected to negative selection in the process of evolution, four genes (*rbc*L, *ndh*F, *rps*8, and *ycf*2) were detected to have undergone positive selection. The *rbc*L gene is responsible for encoding the large subunit of ribulose-1,5-bisphosphate carboxylase/oxygenase (Rubisco), which catalyzes carbon fixation of photosynthesis (Singh et al., 2017). On account of being the target of various environmental selection factors such as drought level, temperature, and carbon dioxide concentration, the *rbc*L gene was usually under positive selection, which is used as the common molecular marker to explore phylogenetic relationships of plants (Wang et al., 2022a; Zhu et al., 2022). The *ndh*F gene encodes subunit protein of nicotinamide adenine dinucleotide (NADH) dehydrogenase complex participating in photosynthetic electron transport. Prior studies indicated that *ndh* genes enabled plants to survive in diverse stressful terrestrial conditions and to maintain efficient photosynthesis (Sabater, 2021). The adaptive evolution of *ndh*F gene could possibly influence the energy transformation and resistance to environmental stresses (Wang et al., 2022a). The *rps*8 gene encodes small ribosomal subunit protein that is involved in protein translation. The exact functional role of the *ycf*2 gene remained unclear. Several studies indicated that the *ycf*2 gene encoded products that were important to chloroplast protein import and cell survival (Kikuchi et al., 2018; Huang et al., 2021).

The classification and phylogenetic relationships of *Uncaria* species have been still under debate (Chen and Taylor, 2011; Zhao et al., 2018). Prior phylogenetic research studies using different molecular markers found that some clades within *Uncaria* showed low support rates (Zhang et al., 2015; Zhu et al., 2018; Liu et al., 2023). For example, Zhang et al. (2015) employed NJ method to reconstruct a phylogenetic tree of *Uncaria* species based on ITS2 sequences, which showed that *U. homomalla* and *U. hirsuta* formed a monophyletic clade with the support rate of 21% (Zhang et al., 2015). In this research, the topologies of the two phylogenetic trees reconstructed by the complete chloroplast genome sequences and common protein-coding sequences showed high similarity, and all the clades within the genus *Uncaria* were well supported. *Uncaria* species appeared to be close to Naucleeae species in our two phylogenetic trees, which was in agreement with prior studies based on DNA molecular markers (Razafimandimbison and Bremer, 2001; Manns and Bremer, 2010). Our results did not support the view of the genus *Uncaira* belonging to Cinchoneae. “*Flora of China*” suggested that further investigation was needed to determine whether *U. scandens* and *U. homomalla* should be regarded as two distinct species due to their high morphological similarity (Chen and Taylor, 2011). *U. homomalla* and *U. scandens* formed a monophyletic clade in our phylogenetic trees. Nonetheless, compared with *U. homomalla*, *U. scandens* lost *ccs*A gene, and their numbers and distributions of SSRs also had differences. Moreover, Zhu et al. (2018) used NJ, MP, and BI methods to construct the phylogenetic trees based on ITS sequence, which indicated that *U. homomalla* and *U. scandens* were not clustered into a monophyletic clade. Here, we agreed that *U. homomalla* and *U. scandens* were two distinct species, but it is necessary to expand the sampling range and sample size to further confirm the reliability of this view. On the basis of the chemical composition and phylogenetic analysis, Zhao et al. (2018) proposed that *U. sinensis* may be a variant of *U. rhynchophylla*. The phylogenetic analysis of ITS or ITS2 sequences also showed that *U. rhynchophylla* and *U. sinensis* formed a monophyletic clade (Zhu et al., 2018; Liu et al., 2023). However, *U. rhynchophylla* and *U. sinensis* were not clustered as a monophyletic clade in the present study. The phylogenetic conflicts may be owing to incomplete lineage sorting, evolutionary rate difference and introgressive hybridization (Zhang et al., 2022). From morphological points of view, the stipules of *U. sinensis* were entire or shallowly emarginate, obviously different from *U. rhynchophylla*, which had deeply bifid stipules. In addition, the diameter of flowering head of *U. sinensis* (12 mm to 15 mm, excluding corolla) was larger than that in *U. rhynchophylla* (4 mm to 8 mm, excluding corolla). The diameter of fruiting head also showed a difference between *U. sinensis* (20 mm to 30 mm) and *U. rhynchophylla* (10 mm to 20 mm). Therefore, *U. sinensis* was not a variant of *U. rhynchophylla* based on phylogenetic analysis of chloroplast genomes and morphological features. Although Ridsdale (1978a) suggested *U. rhynchophylloides* as a synonym of *U. rhynchophylla*, “*Flora of China*” presented that they were two distinct species due to their differences in stipules morphology and diameter of flower head (Chen and Taylor, 2011). Our study also exhibited that *U. rhynchophylloides* and *U. rhynchophylla* were not the same species, as they were located far apart in our phylogenetic trees, which was similar to previous studies based on ITS or ITS2 sequences (Zhu et al., 2018; Liu et al., 2023). In summary, we clearly elucidated the phylogenetic relationships of *Uncaria* species and resolved some taxonomic disputes.

## 5 Conclusion

In this study, the nine chloroplast genomes of *Uncaria* species were highly conserved in structure, gene content, SSR distributions, sequence divergence, and codon usage bias. Ten highly variable loci and four positive selection genes were identified, which provide a reference for further studies on development of specific molecular markers and adaptive evolutionary evaluation of *Uncaria* species. In addition, the phylogenetic relationships of *Uncaria* and closely related species were clearly elucidated. The phylogenetic analysis results based on chloroplast genome showed the genus *Uncaira* belonging to Naucleeae. *U. sinensis* was not a variant of *U. rhynchophylla*. *U. rhynchophylloides* and *U. rhynchophylla* were not the same species. In summary, these findings are helpful for better understanding the evolutionary patterns and phylogenetic relationships of *Uncaria* species.

## Data availability statement

The datasets presented in this study can be found in online repositories. The names of the repository/repositories and accession number(s) can be found in the article/**Supplementary Material**.

## Author contributions

JD: Conceptualization, Data curation, Investigation, Methodology, Supervision, Writing – original draft. QL: Data curation, Investigation, Writing – review & editing. XX: Data curation, Writing – review & editing. ZT: Investigation, Writing – review & editing. YL: Investigation, Writing – review & editing. XG: Funding acquisition, Supervision, Writing – review & editing. SZ: Data curation, Supervision, Validation, Writing – review & editing.
