# Supplementary material for: Comparative and phylogenetic analysis of the complete chloroplast genomes of Uncaria (Rubiaceae) species
Source: Front Plant Sci. 2023 Dec 22;14:1271689. doi: 10.3389/fpls.2023.1271689 (PMC10766718; doi:10.3389/fpls.2023.1271689)
Supplement: Supplementary file 1 [file DataSheet_1.pdf]

Supplementary Table S1 Details of six samples of *Uncaria* species.

Species	Voucher	Accession number	Location	Longitude (E)	Latitude (N)
U. hirsuta	MGT-1	OQ679827	Guangzhou, Guangdong	113°25′18″	23°3′42″
U. homomalla	BYG-1	OQ679828	Guangzhou, Guangdong	113°22′32″	23°11′33″
U. lancifolia	PZY-1	OQ679829	Mengzi, Yunnan	103°42′7″	22°56′60″
U. rhynchophylla	PGT-3	OQ679830	Jianhe, Guizhou	108°25′38″	26°44′21″
U. rhynchophylloides	GSQ-1	OQ679831	Zhaoqing, Guangdong,	112°0′1″	23°11′13″
U. sinensis	HBE-2	OQ679832	Lichuan, Hubei	108°59′55″	30°20′22″

Supplementary Table S2 The chloroplast genomes downloaded from GeneBank database.

Species	GeneBank accession number
Damnacanthus indicus	MW548283
Dunnia sinensis	MN883829
Foonchewia coriacea	MT942688
Galium aparine	KY562587
Galium mollugo	KY562588
Gynochthodes cochinchinensis	MW026443
Hedyotis ovata	MK203877
Kelloggia chinensis	OK236359
Leptodermis forrestii	MT674522
Leptodermis kumaonensis	MT674513
Leptodermis scabrida	MT674519
Morinda officinalis	KR869730
Oldenlandia corymbosa	MT767006
Oldenlandia diffusa	MT767008
Ophiorrhiza densa	MW683127
Ophiorrhiza pumila	MW528277
Paederia foetida	OL449949
Paederia scandens	MN567112
Pseudogalium paradoxum	OK236360
Psychotria rubra	OL679532
Rubia cordifolia	MN736957
Rubia podantha	OM022232
Rubia yunnanensis	OL467345
Saprosma merrillii	MK203879
Calycophyllum spruceanum	OK326865
Coffea arabica	MN894552
Coffea liberica	MW970411
Coffea racemosa	MW970412
Emmenopterys henryi	KY273445
Fosbergia shweliensis	MT180075
Gardenia jasminoides	MZ151501
Gardenia stenophylla	OL517769
Gelsemium sempervirens	MZ440352
Ixora chinensis	MZ221832
Ixora x williamsii	MZ888772
Mussaenda hirsutula	MK203878
Scyphiphora hydrophyllacea	MN390972
Cinchona officinalis	MZ151891
Mitragyna speciosa	KY085908
Neolamarckia cadamba	MG572117
Neolamarckia macrophylla	MN877388
Uncaria scandens	ON243637

Uncaria macrophylla	ON243636
Uncaria sessilifructus	ON243635

Supplementary Table S3 Summary of chloroplast genome sequencing of six *Uncaria* species.

	U. hirsuta	*U. homomalla*	*U. lancifolia*	*U. rhynchophylla*	*U. rhynchophylloides*	*U. sinensis*
Raw data	5.47Gb	4.53Gb	8.69Gb	9.59Gb	11.92Gb	10,24Gb
Raw reads	36,450,078	30,177,278	57,948,224	63,903,980	79,465,584	68,294,536
Clean data	5.36Gb	4.46Gb	8.49Gb	9.41Gb	11.45Gb	10.05Gb
Clean reads	36,128,674	30,006,830	57,613,512	63,316,314	77,441,920	67,463,008
Q20	96.43%	96.68%	97.66%	96.86%	96.23%	96.22%
Q30	90.24%	90.88%	93.12%	91.22%	90.21%	90.25%
Average depth of coverage	1815×	1243×	2163×	3595×	1545×	7934×

Supplementary Table S4 The annotated genes in chloroplast genomes of *Uncaria* species.

Gene group	Gene name
Subunits of photosystem I	*psaA*, *psaB*, *psaC*, *psaI*, *psaJ*
Subunits of photosystem II	*psbA*, *psbB*, *psbC*, *psbD*, *psbE*, *psbF*, *psbH*, *psbI*, *psbJ*, *psbK*, *psbL*, *psbM*, *psbN*, *psbT*, *psbZ*
Subunits of NADH dehydrogenase	*ndhA**, *ndhB**(2), *ndhC*, *ndhD*, *ndhE*, *ndhF*, *ndhG*, *ndhH*, *ndhI*, *ndhJ*, *ndhK*
Subunits of cytochrome b/f complex	*petA*, *petB**, *petD**, *petG*, *petL*, *petN*
Subunits of ATP synthase	*atpA*, *atpB*, *atpE*, *atpF**, *atpH*, *atpI*
Large subunit of rubisco	*rbcL*
Proteins of large ribosomal subunit	*rpl14*, *rpl16**, *rpl2**(2), *rpl20*, *rpl22*, *rpl23*(2), *rpl32*, *rpl33*, *rpl36*
Proteins of small ribosomal subunit	*rps11*, *rps12***(2), *rps14*, *rps15, rps16**, *rps18*, *rps19*, *rps2*, *rps3*, *rps4*, *rps7*(2), *rps8*
Subunits of RNA polymerase	*rpoA*, *rpoB*, *rpoC1**, *rpoC2*
Ribosomal RNAs	*rrn16*(2), *rrn23*(2), *rrn4.5*(2), *rrn5*(2)
Transfer RNAs	*trnA-UGC**(2), *trnC-GCA*, *trnD-GUC*, *trnE-UUC, trnF-GAA*, *trnG-GCC*, *trnG-UCC**, *trnH-GUG*, *trnI-CAU*(2), *trnI-GAU**(2), *trnK-UUU**, *trnL-CAA*(2), *trnL-UAA**, *trnL-UAG*, *trnM-CAU, trnN-GUU*(2), *trnP-UGG*, *trnQ-UUG*, *trnR-ACG*(2), *trnR-UCU, trnS-GCU*, *trnS-GGA*, *trnS-UGA*, *trnT-GGU*, *trnT-UGU*, *trnV-GAC*(2),*trnV-UAC**, *trnW-CCA*, *trnY-GUA*, *trnfM-CAU*
Maturase	*matK*
Protease	*clpP***
Envelope membrane protein	*cemA*
Acetyl-CoA carboxylase	*accD*
c-type cytochrome synthesis gene	*ccsA*
Translation initiation factor	*infA*
Conserved open reading frames	*ycf1*, *ycf2*(2), *ycf3***, *ycf4*

Note: * indicates gene with on intron; ** indicates gene with two introns; (2) indicates two copies gene.

Supplementary Table S5 The interspecific genetic distance analysis based on complete chloroplast genome sequences of *Uncaria* species

	U. hirsuta	*U. homomalla*	*U. lancifolia*	*U. macrophylla*	*U. rhynchophylla*	*U. rhynchophylloides*	*U. scandens*	*U. sessilifructus*
U.homomalla	0.0018							
U. lancifolia	0.0059	0.0059						
U.macrophylla	0.0058	0.0058	0.0042					
U. rhynchophylla	0.0019	0.0018	0.0060	0.0058				
U. rhynchophylloides	0.0042	0.0041	0.0043	0.0044	0.0043			
U. scandens	0.0016	0.0009	0.0057	0.0056	0.0016	0.0040		
U. sessilifructus	0.0062	0.0062	0.0046	0.0037	0.0062	0.0047	0.0060	
U. sinensis	0.0048	0.0046	0.0060	0.0060	0.0049	0.0044	0.0045	0.0064

Supplementary Table S6 The interspecific genetic distance analysis based on common protein coding sequences of chloroplast genome in *Uncaria* species

	U. hirsuta	*U. homomalla*	*U. lancifolia*	*U. macrophylla*	*U. rhynchophylla*	*U. rhynchophylloides*	*U. scandens*	*U. sessilifructus*
U.homomalla	0.0013							
U. lancifolia	0.0041	0.0042						
U.macrophylla	0.0039	0.0041	0.0032					
U. rhynchophylla	0.0014	0.0013	0.0043	0.0041				
U. rhynchophylloides	0.0029	0.0030	0.0034	0.0035	0.0031			
U. scandens	0.0011	0.0007	0.0040	0.0039	0.0012	0.0028		
U. sessilifructus	0.0044	0.0046	0.0036	0.0032	0.0046	0.0038	0.0043	
U. sinensis	0.0034	0.0034	0.0047	0.0047	0.0036	0.0036	0.0033	0.0052

Supplementary Table S7 Correlation analysis of codon usage bias parameters.

Species		GC1	GC2	GC12	GC3	GC3s
U. hirsuta	GC2	0.432**				
	GC12	0.859**	0.833**			
	GC3	0.167	0.124	0.173		
	GC3s	0.172	0.116	0.172	0.953**	
	ENC	0.199	-0.288	-0.007	0.490**	0.482**
U. homomalla	GC2	0.425**				
	GC12	0.857**	0.831**			
	GC3	0.176	0.106	0.169		
	GC3s	0.174	0.091	0.159	0.953**	
	ENC	0.195	-0.238	-0.015	0.506**	0.497**
U. lancifolia	GC2	0.417**				
	GC12	0.855**	0.828**			
	GC3	0.177	0.115	0.175		
	GC3s	0.187	0.103	0.174	0.954**	
	ENC	0.229	-0.245	0.002	0.466**	0.448**
U. macrophylla	GC2	0.414**				
	GC12	0.854**	0.827**			
	GC3	0.168	0.127	0.176		
	GC3s	0.172	0.114	0.172	0.953**	
	ENC	0.228	-0.214	0.019	0.490**	0.490**
U. rhynchophylla	GC2	0.427**				
	GC12	0.862**	0.826**			
	GC3	0.152	0.093	0.147		
	GC3s	0.158	0.081	0.144	0.954**	
	ENC	0.195	-0.242	-0.012	0.495**	0.488**
U. rhynchophylloides	GC2	0.422**				
	GC12	0.854**	0.832**			
	GC3	0.141	0.088	0.137		
	GC3s	0.148	0.08	0.136	0.953**	
	ENC	0.199	-0.247	-0.02	0.482**	0.477**
U. scandens	GC2	0.429**				
	GC12	0.858**	0.832**			
	GC3	0.164	0.115	0.166		
	GC3s	0.17	0.103	0.163	0.953**	
	ENC	0.205	-0.227	-0.003	0.502**	0.491**
U. sessilifructus	GC2	0.417**				
	GC12	0.860**	0.822**			
	GC3	0.169	0.095	0.159		
	GC3s	0.183	0.087	0.163	0.953**	
	ENC	0.153	-0.25	-0.045	0.485**	0.479**

	GC2	0.430**				
	GC12	0.858**	0.833**			
U. sinensis	GC3	0.192	0.155	0.206		
	GC3s	0.196	0.14	0.2	0.949**	
	ENC	0.236	-0.209	0.026	0.470**	0.467**

Note: * indicates significant difference at 0.05 level; ** indicates significant difference at 0.01 level.

Supplementary Table S8 Positive selection sites identified by site model.

Gene	Model	LnL	LRT p-value	Positive sites
ndhF	M3	-3433.459589	M0 vs.M3: 0.000202026	508 S 0.990*
	M0	-3444.450882	M1a vs.M2a: 0.008373251	
	M2a	-3433.549675	M7 vs.M8: 0.007448438	
	M1a	-3438.332388	M8a vs.M8: 0.002101071	
	M8	-3433.602846		
	M7	-3438.502597		
	M8a	-3438.332383		
rbcL	M3	-2085.601310	M0 vs.M3: 0.000104968	40 P 0.963*
	M0	-2097.305059	M1a vs.M2a: 0.000131994	237 T 0.956*
	M2a	-2085.601306	M7 vs.M8: 0.000131815	256 E 0.956*
	M1a	-2085.601306	M8a vs.M8: 0.000023708	258 M 0.962*
	M8	-2085.601305		286 T 0.964*
	M7	-2094.535418		
	M8a	-2094.534049		
rps8	M3	-560.989593	M0 vs.M3: 0.003180868	77 F 0.980*
	M0	-568.930873	M1a vs.M2a: 0.008557904	
	M2a	-560.989593	M7 vs.M8: 0.011508735	
	M1a	-565.750493	M8a vs.M8: 0.002583132	
	M8	-561.210108		
	M7	-565.674757		
	M8a	-565.750486		
ycf2	M3	-9385.483706	M0 vs.M3: 0.000006246	1263 L 0.996**
	M0	-9400.223485	M1a vs.M2a: 0.000008020	2082 C 0.995**
	M2a	-9385.484307	M7 vs.M8: 0.000000682	
	M1a	-9397.217835	M8a vs.M8: 0.000001274	
	M8	-9385.487142		
	M7	-9399.685919		
	M8a	-9397.217822		

Note: * indicates that the posterior probabilities of the site are > 0.95; ** indicates that the posterior probabilities of the site are > 0.99

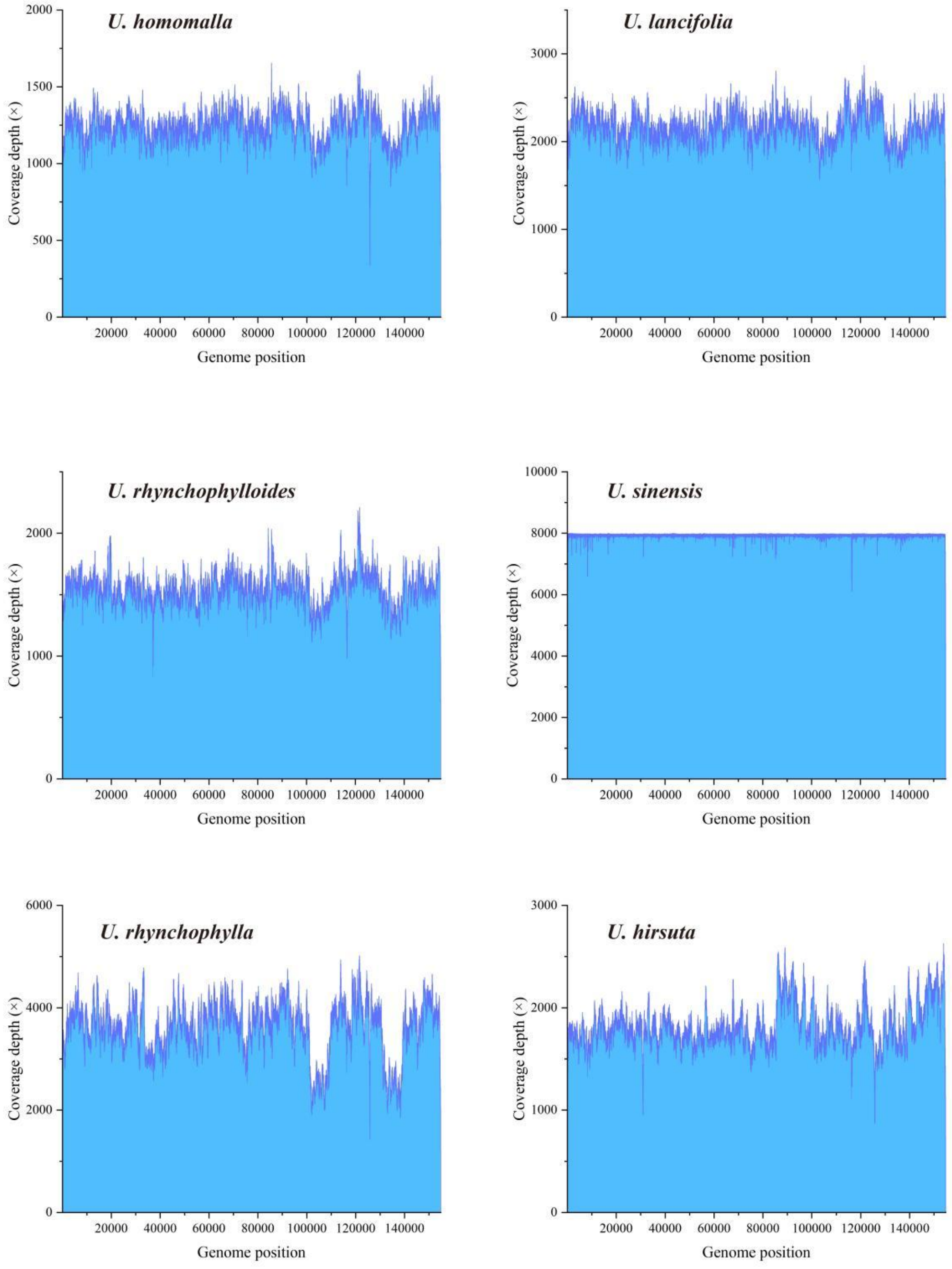

Supplementary Figure S1 The coverage depths of each position of chloroplast genome of *Uncaria* species.

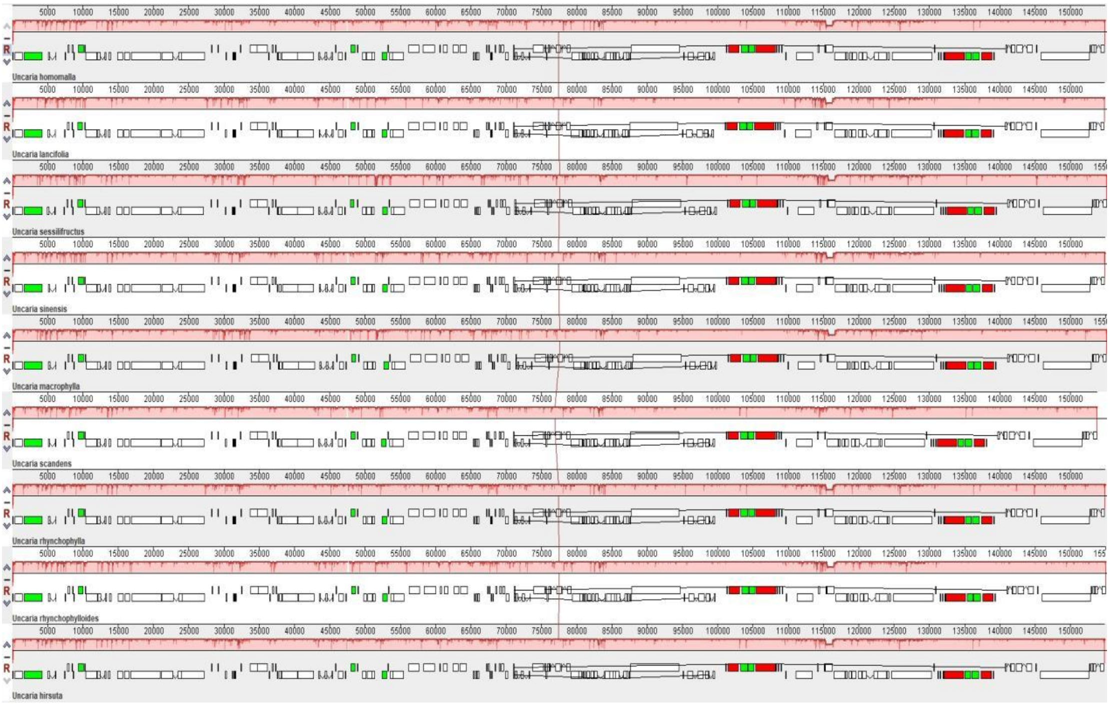

Supplementary Figure S2 Mauve alignment of nine chloroplast genomes of *Uncaria* species.

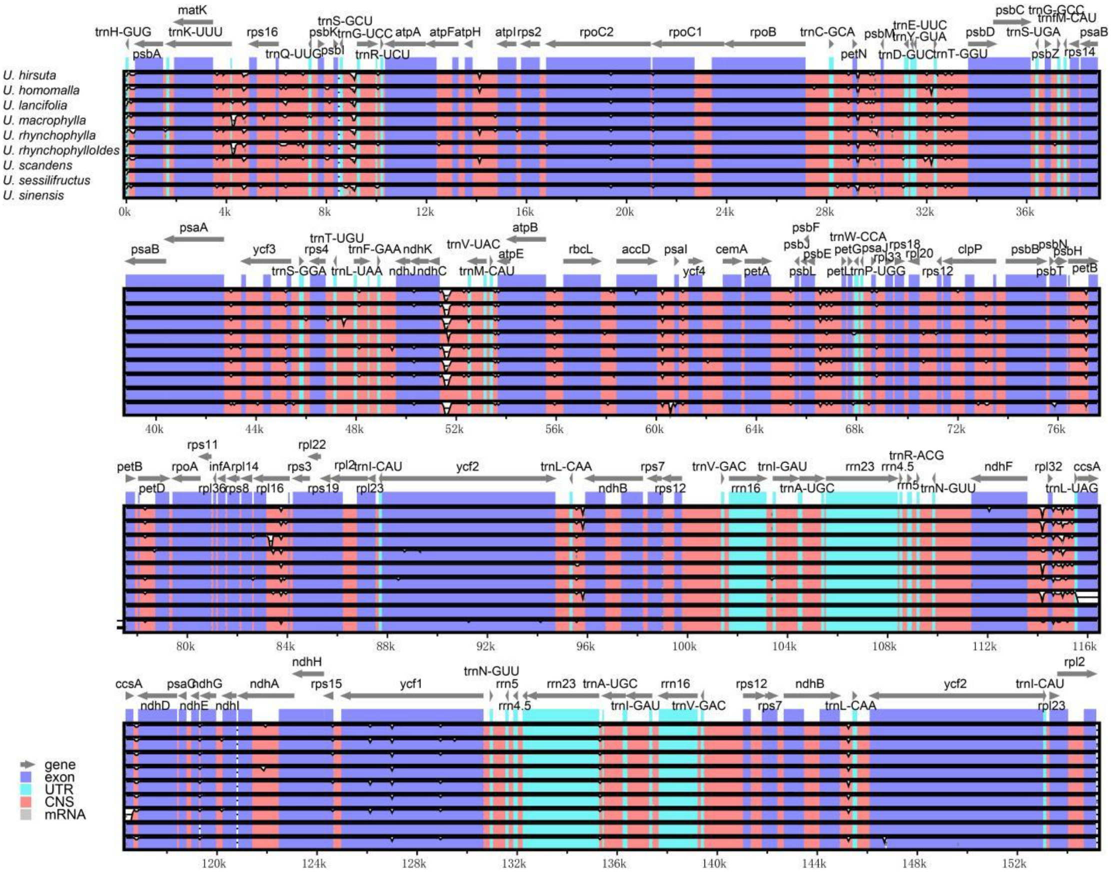

Supplementary Figure S3 Comparison of chloroplast genomes of nine *Uncaria* species using mVISTA.

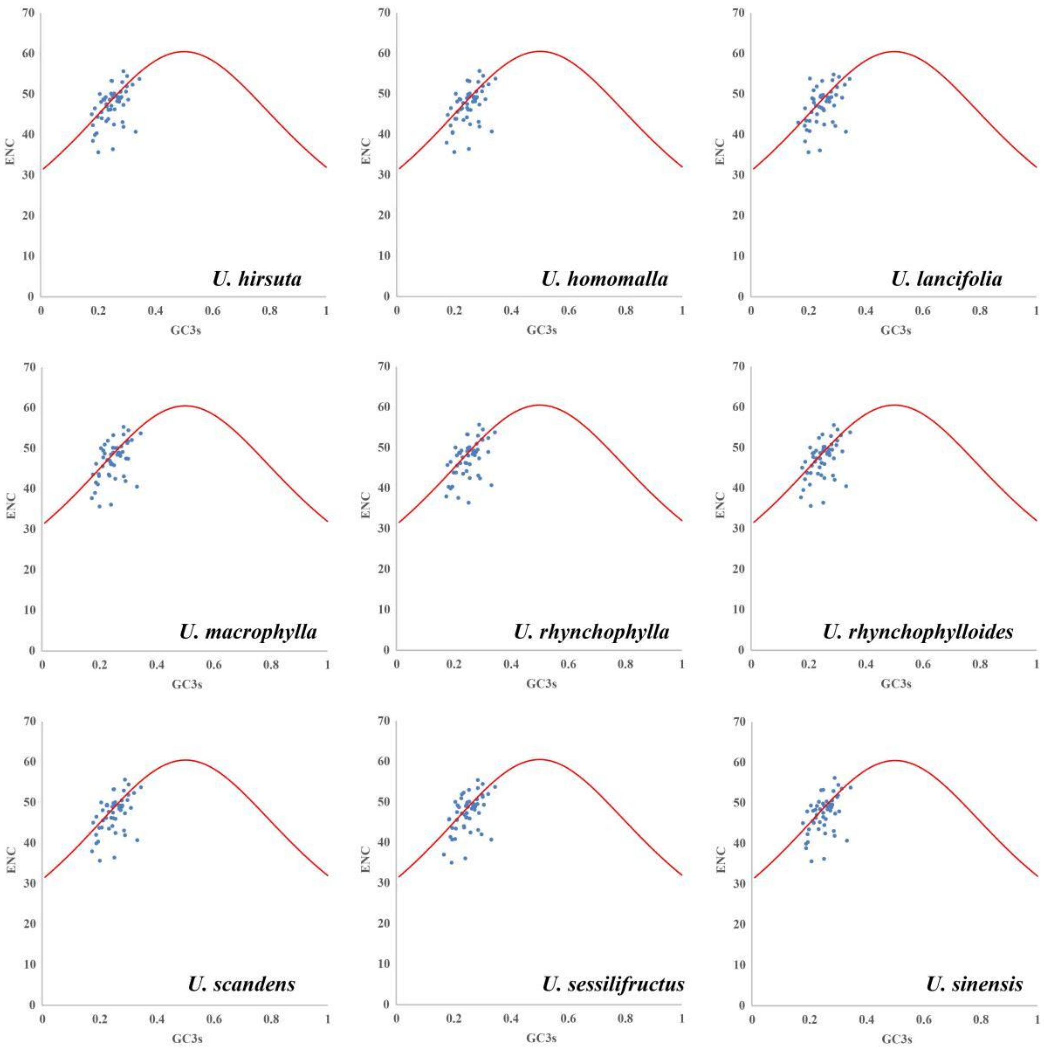

Supplementary Figure S4 ENC-plot analysis.

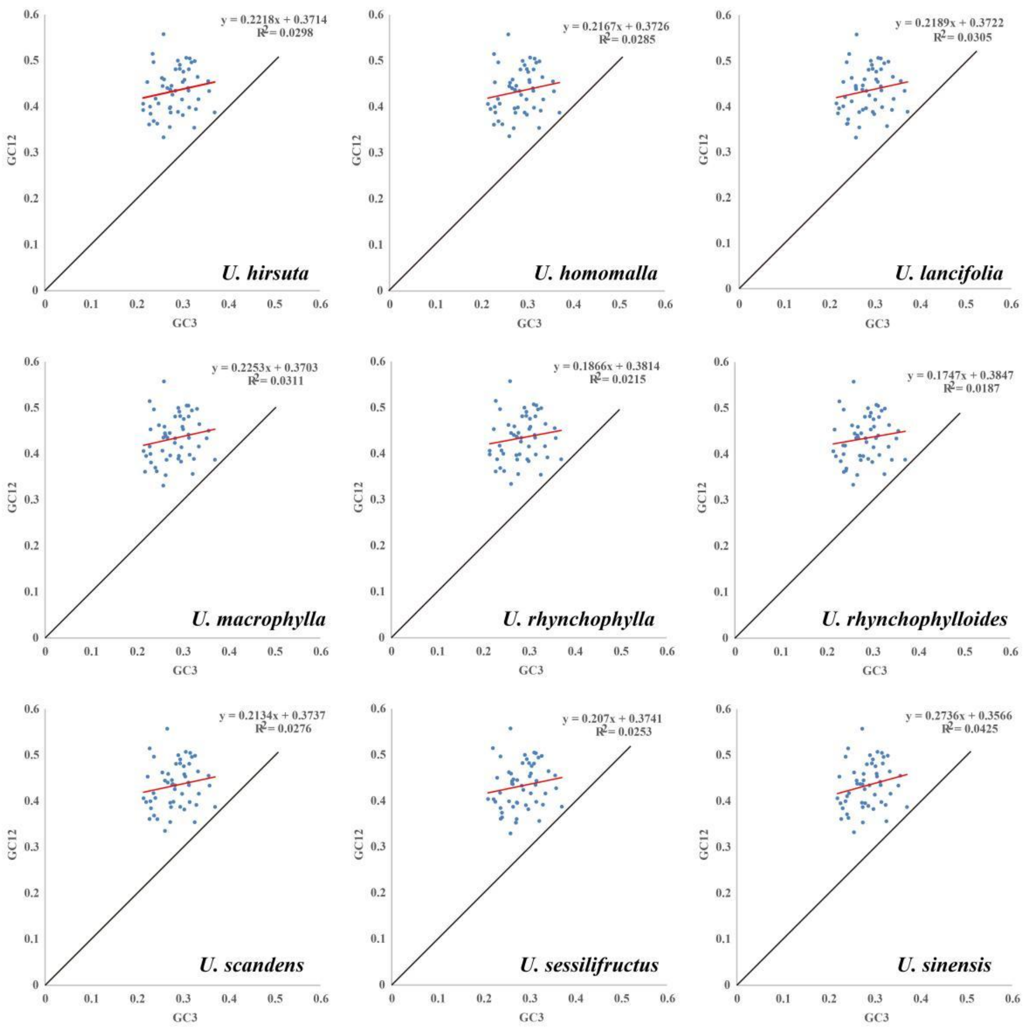

Supplementary Figure S5 Neutrality plot analysis.

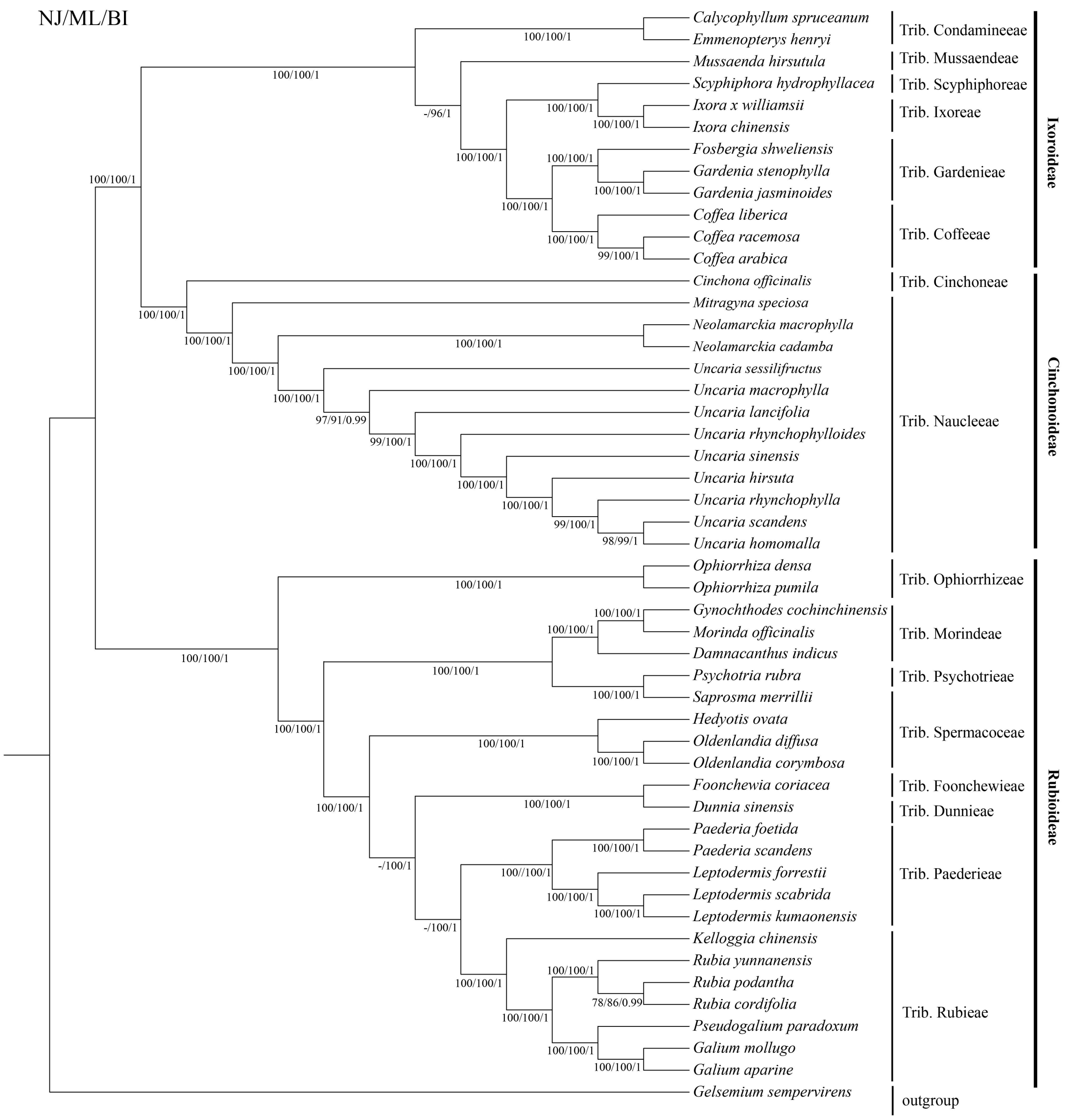

Supplementary Figure S6 Phylogenetic tree of *Uncaria* and closely related species based on the common protein coding sequences of chloroplast genome.
